# The role of controllable lifestyle in the choice of specialisation among Hungarian medical doctors

**DOI:** 10.1186/s12909-017-1031-z

**Published:** 2017-11-13

**Authors:** Edmond Girasek, Miklós Szócska, Eszter Kovács, Péter Gaál

**Affiliations:** Health Services Management Training Centre, Semmelweis University, Kútvölgyi út 2, Budapest, 1125 Hungary

**Keywords:** Medical students, Medical doctors, Specialisation, Controllable lifestyle, Career choice

## Abstract

**Background:**

Hungary has been serious facing human resources crisis in health care, as a result of a massive emigration of health workers. The resulting shortage is unevenly distributed among medical specialisations. The findings of research studies are consistent in that the most important motivating factor of the choice of the medical career and of medical specialisations is professional interest. Beyond this, it is important to examine other reasons of why students do or do not choose certain specialisations. The lifestyle determined by the chosen speciality is one such factor described in the literature.

**Methods:**

Using convenient sampling, first year resident medical doctors from each of the four Hungarian universities with a medical faculty were asked to participate in the study in 2008. In total 391 first year resident medical doctors completed the self-administered questionnaire indicating a 57.3% response rate. On the basis of the work of Schwartz et al. (Acad Med 65(3):207–210, 1990), the specialisation fields were divided into the two main categories of non-controllable (NCL) or controllable lifestyles (CL). We carried out a factor analysis on motivating factors and set up an explanatory model regarding the choice of CL and NCL specialisations.

**Results:**

Two maximum likelihood factors were extracted from the motivational questions: “lifestyle and income” and “professional interest and consciousness”. The explanatory model on specialisation choice shows that the “professional interest and consciousness” factor increases the likelihood of choosing NCL specialisations. In contrast the “lifestyle and income” factor has no significant impact on the choice of CL/NCL specialisations in the model.

**Conclusions:**

Our results confirm the important role of professional interest in the choice of medical specializations in Hungary. On the other hand, it seems surprising that we found no significant difference in the “lifestyle and income” related motivation among those medical residents, who opted for CL as opposed to those, who opted for NCL specialisations. This does not necessarily mean that lifestyle is not an important motivating factor, but that it is equally important for both groups of medical residents.

**Electronic supplementary material:**

The online version of this article (10.1186/s12909-017-1031-z) contains supplementary material, which is available to authorized users.

## Background

The availability of sufficient human resources with the appropriate skill mix is a major challenge of contemporary health systems [1]. Hungary well exemplifies this challenge, as it has been facing a serious human resources crisis, which threatens the sustainability of the health system [2, 3]. While during the communist regime Hungary had a surplus of physicians, the number of medical doctors registered to practice per 100,000 population fell below the EU-27 average by 11% in 2005, and has remained there since then [4]. The loss of practising physicians is mainly attributable to migration. Since 2004, when Hungary joined the EU, more than 10,000 medical doctors have applied for the diploma certification needed to work abroad [5], representing one-third of the active medical workforce. Moreover, as young doctors are generally more mobile, the remaining population of physicians is aging. In 2014 55.0% of the practising medical doctors were 50 years old or older, and it is projected that this number will increase to 64.4% by 2021, if the current trend continues [6].

This is further aggravated by the inequitable distribution of the remaining doctors, both by specialisations and geographic location. According to the data of the Hungarian Central Statistical Office, the difference between the counties with the highest and lowest density of medical doctors was as high as 2.5 fold in 2014, even if we exclude Budapest, the capital of Hungary [7]. Further, the number of so-called “shortage specializations” is increasing, where it is not possible to recruit enough medical doctors to fill in the vacancies of health care providers, despite the extra compensation offered by the government. In 2016, shortage specializations included, among others, radiology, pathology, psychiatry, general and vascular surgery. From the policy perspective, therefore, it is of crucial importance for Hungary to understand the factors influencing medical career choices, including, but not limited to, the choice of specializations.

Medical career choices have been brought into the focus of research for several years, first in the USA and then in other countries. The findings are consistent that the most important motivation for the choice of the medical profession and of specialisations is professional interest, followed by the professional and scientific challenges, interpersonal, doctor-patient relations, and social aspects like income, work burden, life-style and prestige [8–11], with factors related to quality of life getting increasing attention [12].

The lifestyle determined by the chosen speciality was first mentioned in 1989 by Schwartz et al. [13], who found that an increasing number of medical doctors chose those specialisations, where the working time could be well planned. In 1990 Schwartz and his colleagues assessed 4th grade medical students by a 25-item questionnaire focusing on their choice of specialisation [14]. In the frame of a factor analysis, they combined the response items into three motivation factors: “perceived lifestyle”, “professional challenges and practice-orientation”, and “altruistic values and attitudes”. “Perceived lifestyle” (income, free-time and prestige) was the strongest determining factor in the choice of specialisation. They created two groups, controllable lifestyle (CL) and non-controllable lifestyle (NCL) specialisations, on the basis of whether or not the medical doctor could control work hours, and found a significant correlation between the motivation patterns and the choice of CL or NCL specialities.

In 2003, Lind and Cendan [15] coined the term “lifestyle-friendly” specialisations and found them chosen more frequently in the USA during the period of 1982–2002. This increase was significantly higher among women indicating that they valued more the “lifestyle-friendly” features of the job than men. Regarding trends, other researchers, such as Dorsey et al. [16] and Lambert et al. [17], arrived at the same conclusion. While Newton et al. also found lifestyle as becoming more important to medical students in their career choice, they emphasised income as another crucial factor, and the relative influence of these two factors varied considerably between specialties [18]. In the UK, Smith et al. found similar results in 2015: work-life balance became more important for junior doctors’ choice of speciality [19].

Based on the previous findings, this study aims to explore the importance of lifestyle in the choice of medical specialisation in Hungary, using the CL-NCL categorisation of Schwartz et al. [14], to support policy-making in ensuring a more equitable distribution of specialists all over the country. To our knowledge, no such research has been carried out so far in Hungary.

## Methods

### Participants and procedure

Using convenient sampling, resident medical doctors from each of the four Hungarian universities with a faculty of general medicine were asked to participate in the survey.

A resident medical doctor or medical resident (in Hungarian “rezidens”) is a person, who has already completed undergraduate medical education, and has been admitted to a centrally organized two-year postgraduate training program in one of the medical specialisations. At the time of our study, these positions were centrally financed, while the admission procedure, the program and the examinations were administered by one of the four Hungarian universities with a medical faculty. Residents practice medicine under the supervision of fully licensed specialists, usually in clinical departments of the university concerned or in teaching hospitals.

The self-administered questionnaire survey was carried out during the residency training. The questionnaire was distributed at the time of the final written test exams of a mandatory course for all resident doctors, as part of the exam package. One member of the research team briefly introduced the objectives of the study and explained that participation was voluntary and anonymous. In total 391 resident medical doctors in their first year of study completed the questionnaire indicating a 57.3% response rate. In 2008 the government offered in total 682 new medical resident posts, whose 96.2% was actually filled.

We opted for convenient sampling, because there has been no list of all of the Hungarian resident doctors available, which could have been used as a sampling frame. It is important to note, that we could not achieve the total population sample, to a smaller extent because of refusals, but mainly because first year medical residents have been offered the choice of completing the mandatory course either in the first year or in the second year of their residency training. Due to the lack of statistical data on the study population, no comparison of our final sample was possible. Nevertheless, there has been no indication of sampling bias and the response rate suggests that this survey represents the valid motivations and opinion of first year medical residents in Hungary.

### Measurement instruments

Residents completed a questionnaire containing different sections on career plans. They were asked about their motivations to become a medical doctor, the factors determining their choice of medical specialisation, and about their intention to work abroad. In this paper we focus solely on analysing the responses concerning the motivations on specialisation choice.

Although Rogers and colleagues [20] were in the process of developing a questionnaire on career choices and motivations, there was no internationally validated measurement instrument on specialisation choice at the time of our research, so we developed our own questionnaire.

We put together the set of questions and items about specialisation choices on the basis of the review of international and national literature and a qualitative study [14, 21, 22]. Twelve medical residents were interviewed to explore personal experiences for developing the questions. Based on these findings, the questionnaire was piloted with 10 medical residents, who belonged to the target group. According to their comments and the pilot-test experiences, the tool was finalised. This fine-tuning in most of the cases involved interpretation issues and grammatical corrections. (The questionnaire can be found in the Additional file 1.)

The motivations of specialisation choice were examined retrospectively [23], where the respondents rated 14 items (e.g. “This is my interest”, “Good job opportunities”, “Lifestyle”) on a five-point Likert-scale. We tested the internal consistency of the specialisation choice motivation questionnaire, and found it appropriate (Cronbach-alpha = .729) [24].

We identified the respondents chosen field of specialisation on the basis of the question “Which specialisation programme do you take part in order to obtain specialisation?” During the analysis the specialisation fields were grouped into two main categories: non-controllable (NCL) or controllable lifestyles (CL), based on Schwartz et al. [14]. The NCL specialities included internal medicine, family medicine, paediatrics, obstetrics-gynaecology, general surgery and surgical specialities, while CL specialities were anaesthesiology, dermatology, emergency medicine, neurology, ophthalmology, otolaryngology, pathology, psychiatry and radiology.

### Statistical analysis

Non-weighted data were analysed using the SPSS 20.0 software. In the statistical analysis descriptive statistics, frequencies, crosstabs and means, were calculated. In line with Allen and Seaman [25], who argue that a Likert-scale can be considered an interval variable, if the interval is the attribute of the data (there are numerical values) and has at least 5 categories, and similarly to other papers of the study [11, 26], we analysed the five scale Likert-scale questions as interval variables, calculating their means and standard deviations. The next step was confirmatory factor analysis, Maximum likelihood with Varimax rotation. Finally, binary logistic regression tests were carried out to build an explanatory model for the lifestyle of the given speciality (NCL or CL), where the independent variables were the extracted specialisation choice factors.

We assumed that those respondents, who opted for CL specialties would rate lifestyle-related specialization choice factors higher, and there would be a statistically significant difference between the two groups of medical residents in this respect.

## Results

Table 1 shows the basic socio-demographic features, namely, the gender and age distribution of respondents. Over two-thirds (68.3%) of the respondent first year resident doctors were female and 31.7% male. Regarding the age distribution, only 13.8% of the respondents were 30 years old or over, while the 86.2% majority was divided roughly equally among the 24–26 and 27–29 age groups. The geographical distribution shows that 51.2% of first year students were from Semmelweis University, Budapest, 22.5% from the University of Debrecen, 16.5% from the University of Pécs and 9% from the University of Szeged.

**Table 1:** Gender and age distribution of respondents

	24–26 years	27–29 years	30 years or more	Total
Male	46	54	17	117
	39.3%	46.2%	14.5%	100.0%
Female	119	99	34	252
	47.2%	39.3%	13.5%	100.0%
Total	165	153	51	369
	44.7%	41.5%	13.8%	100.0%

### Descriptive statistics of specialisation choice

Table 2 provides a simple descriptive statistics of the answers for question D3 of the questionnaire, including the mean, standard deviation and standard error for each response items. The question and the predefined motivational items, which were to be rated on a five-level rating scale, are presented in accordance with the original questionnaire. The only difference is that order of the response items has been changed, the results are shown in a decreasing order of importance, while the numbering of the items indicates the order of items in the original questionnaire. This numbering is used consistently throughout the presentation of other findings.

**Table 2:** Mean, standard deviation and standard error of specialisation choice motivation items, derived from question D3 of the questionnaire (decreasing order of importance, the numbering of the items indicates the order of items in the questionnaire)

D3	Please evaluate the following items according to the extent they influenced your choice of specialization (1 = not at all, 5 = definitive influence)!	Mean	Standard deviation	Standard error
1	Focus of my interest	4.26	1.02	0.05
2	Professional challenges	3.82	1.16	0.06
13	Relation with patients	3.55	1.37	0.06
9	Lifestyle	2.83	1.42	0.06
11	Innovation opportunities	2.68	1.39	0.06
14	Good job opportunities	2.65	1.22	0.04
12	Social prestige	2.51	1.26	0.06
7	Opportunities to work abroad	2.41	1.34	0.07
5	Prestige among medical doctors	2.35	1.20	0.07
3	Scientific career	2.10	1.17	0.07
4	It just happened this way	2.10	1.47	0.07
10	Salary	1.96	1.11	0.08
8	Family influence	1.84	1.16	0.07
6	Informal payment	1.44	0.85	0.06

Table 3 shows the mean values for each item separately for male and female respondents. Items, where statistically significant differences can be observed (at *p* < 0.05), are marked with an asterisk. Three items were rated significantly higher by men (“prestige among medical doctors”, “informal payment”, “salary”), while two items by women (“lifestyle”, “relation with patients”). These differences changed the rank order only in the case of two of these five items: “lifestyle” occupies the 4th place among women as opposed to the 7th place among men, while “prestige among medical doctors” is the 9th among women, while the 8th among men.

**Table 3:** Comparison of the means of specialisation choice motivation items by gender (the significant differences, at *p* < 0.05, marked with asterisk, the numbering of items indicates the order of items in the questionnaire)

		Male	Female
1	Focus of my interest	4.26	4.26
2	Professional challenges	3.86	3.81
13	Relation with patients*	**3.23**	**3.73**
11	Innovation opportunities	2.84	2.61
14	Good job opportunities	2.78	2.58
12	Social prestige	2.68	2.45
**9**	Lifestyle*	**2.61**	**2.93**
**5**	Prestige among medical doctors*	**2.57**	**2.25**
7	Opportunities to work abroad	2.52	2.34
4	It just happened this way	2.16	2.07
3	Scientific career	2.14	2.08
10	Salary*	**2.12**	**1.87**
8	Family influence	1.95	1.79
6	Informal payment*	**1.65**	**1.34**

### Specialisations choice - factor analysis

The Maximum likelihood factors were extracted from the motivational questions on specialisation choice. Table 4 shows the extracted factor structure. The first factor, which we named “lifestyle and income”, consists of the following variables: “salary” (0.769), “social prestige” (0.647), “good job opportunities” (0.634), “prestige among medical doctors” (0.528), “lifestyle” (0.548), “informal payment” (0.484), “opportunities to work abroad” (0.465). The second factor, which we named “professional interest and consciousness” consists of the variables of “focus of interest” (0.999), “professional challenges” (0.662) and “it just happened this way” (−0.715).

**Table 4:** Maximum likelihood factors on specialisation choice

	Variables	ML factor 1	ML factor 2
		Lifestyle and income	Professional interest and consciousness
1	Focus of my interest	−0.030	**0.999**
2	Professional challenges	0.129	**0.662**
4	It just happened this way	0.086	**−0.715**
5	Prestige among medical doctors	**0.528**	0.098
6	Informal payment	**0.484**	−0.129
7	Opportunities to work abroad	**0.465**	−0.049
9	Lifestyle	**0.548**	0.045
10	Salary	**0.769**	−0.002
12	Social prestige	**0.647**	0.132
14	Good job opportunities	**0.634**	−0.027

### Motivation factors and gender differences

We compared the mean of the maximum likelihood factors in terms of gender differences, and found that men had a significantly higher value on the “lifestyle and income” factor (Table 5), i.e. their choice of specialization are influenced more by the motivational factors aggregated in this category. This finding is in line with the gender differences observed before insofar as 4 out of the 5 statistically significant differences were experienced in the case of those variables, which came under the “lifestyle and income” factor.

**Table 5:** Mean value of Maximum likelihood factors on specialisation choice motivation – breakdown by gender (significant difference is marked with asterisk)

	Lifestyle and income*	Professional interest and consciousness
Male	0.1552533	0.0065065
Female	−0.0739347	0.0087769

### The association of motivation factors with the controllability of the lifestyle of the chosen speciality

We set up the explanatory model in three steps (see Table 6). The first model examined the relationship between the two factors and the choice of CL versus NCL specialisations. This explanatory model on specialisation choice shows that the “professional interest and consciousness” factor has significant impact on the CL/NCL specialisation choice (OR = 1.534, *p* < 0.000), so higher scores on motivations belonging to the “professional interest and consciousness” factor increase the likelihood of choosing non-controllable-lifestyle specialisations. The Nagelkerke R-square, which represented the goodness-of-fit of the model, was 0.035. On the other hand the “lifestyle and income” factor had no significant impact on CL/NCL specialisation choice in the model (OR = 1.150; *p* > 0.05).

**Table 6:** Binary logistic regression – explanatory models for CL/NCL specialisation choice

	B	S.E.	Wald	df	Sig.	Odds ratio (OR)
Model 1						
Lifestyle and income	0.140	0.122	1.315	1	0.251	1.150
Professional interest and consciousness	0.428	0.120	12.671	1	0.000	1.534
Constant	−0.216	0.110	3.863	1	0.049	0.806
Model 2 (with gender included)						
Lifestyle and income	0.110	0.124	0.786	1	0.375	1.116
Professional interest and consciousness	0.426	0.121	12.425	1	0.000	1.531
Gender	−0.362	0.236	2.349	1	0.125	0.697
Constant	0.403	0.409	0.972	1	0.324	1.497
Model 3 (with gender and age included)						
Lifestyle and income	0.148	0.126	1.394	1	0.238	1.160
Professional interest and consciousness	0.434	0.123	12.421	1	0.000	1.544
Gender	−0.412	0.242	2.892	1	0.089	0.663
Age	0.045	0.053	0.721	1	0.396	1.046
Constant	−0.752	1.535	0.240	1	0.624	0.471

In the second model, we included gender, and in the third model age, as well, as relevant background factors (given that we had a population, which were homogenous in terms of education and occupation, and we had no data on income) and looked at how it altered the results of the first model. As it can be seen in Table 6 the odds ratios were changed only slightly, and the conclusion remained the same. Nevertheless, the Nagelkerke R-square increased to 0.064 with gender included and further to 0.073, with gender and age included, which implies the predictive power of the second model is higher and the third model is the highest.

## Discussion

In the era of technological revolution in medicine accompanied by the increasing need of more and more specialized workforce, investigating the motivations of specialisation choice can be considered an even more important research topic, than the choice of the medical profession itself. Knowing why medical graduates do or do not choose specific specialisations could inform policy making aimed at a more equitable distribution of the medical workforce in a country. Hungary and other countries with a health workforce crisis are especially in need for such research to understand, where should be intervened in the health system and how.

The relationship between lifestyle and medical specialisation choice was first raised by Schwartz et al. in 1989 [13]. They provided an interesting theoretical framework of mapping medical specialisation choice [14], which was used as the basis of our study. According to the findings of Schwartz et al., we also categorized the specialities into two groups, first, where the lifestyle was under the control of the specialist (CL specialities) and second, where it was not (NCL specialities). Our study found two complex motivational factors, confirming a similar factor structure to that of Schwartz et al. The first factor of Schwartz et al., “perceived lifestyle”, by and large covers the same motivational variables (income, free-time and prestige) as our first factor “lifestyle and income” (salary, informal payment; lifestyle; social prestige, prestige among medical doctors). The exception is job opportunities, including opportunities to work abroad, which is partly a Hungarian special related to the recent trend of the emigration of medical doctors mainly to more affluent member states of the EU. The second factor in the model of Schwartz et al., “professional challenges and practice-orientation” overlaps with our “professional interest and consciousness” factor. The other difference between our model and the model of Schwartz et al., is a third factor, “altruistic values and attitudes”, which was not confirmed by the Hungarian data.

Previous studies [14–18] that found an increase in choosing “lifestyle-friendly” specialisations, underlined the importance of lifestyle-related factors in specialisation choice. Given that our study did not look at the trends, we could detect the increased significance of “lifestyle and income” only indirectly, in that the factor analysis produced a separate motivational factor for related motivational items (i.e. salary, social prestige, good job opportunities, prestige among medical doctors, lifestyle, informal payment, opportunities to work abroad).

Our binary logistic regression explanatory model on specialisation choice showed that only the “professional interest and consciousness” factor had significant impact on the choice of CL/NCL specialisations and not the “lifestyle and income” factor. Although unexpected, this does not necessarily mean that lifestyle is not an important motivating factor, but that it is equally important for medical residents choosing the CL as well as for those choosing NCL specialisations. This would suggest that policy makers should focus more on those life-style related factors, which are amenable to policy making. It is also possible that the original USA categorisation of specialisations does not fully fit the Hungarian health system and needs to be refined.

In summary, we managed to confirm the existence of two complex motivation factors in Hungary, similar to the model of Schwartz et al., and found the same significant association between the “professional interest and consciousness” factor and the choice of NCL specialisations as Schwartz et al. did. The apparent lack of significant association between the “lifestyle and income” factor and the choice of CL specialisations needs to be studied further, though, before any strong conclusions can be drawn regarding its real influence on medical specialisation choice.

## Conclusions

The phenomenon of specialisation choice is a topic with high importance in health policy and health education. In our research we studied the topic in the framework published by Schwartz et al. [14], and found many similarities regarding the motivations of specialisation choice of Hungarian and USA medical residents, but also a few notable differences. While our study confirmed the importance of two complex motivation factors, the “professional interest and consciousness” and the “lifestyle and income” factors, it did not do in the case of the third factor of Schwartz et al. [14]. Further, while the “professional interest and consciousness” factor and the choice of NCL specialisations showed a significant association, the same was not true for the “lifestyle and income” factor and the choice of CL specialisations. This could be explained by not just its low importance, but also by an equally high importance in both groups of medical residents, which underlies the need for further research and clarification.

In order to decrease the inequalities across specialisations by addressing the shortages of professionals in certain specialties, health policy decision-makers should monitor the main motivations of medical doctors and the changes over time. Interventions regarding income and working conditions might make certain specialties more attractive, and could ultimately contribute to a better performing health care system.

## Additional file

Additional file 1: Human Resource Research in Healthcare – Questionnaire. (PDF 224 kb)

## Abbreviations

CL: Controllable lifestyle
EU: European Union
NCL: Non-controllable lifestyle
USA: United States of America
