# Supplementary material for: The role of controllable lifestyle in the choice of specialisation among Hungarian medical doctors
Source: BMC Med Educ. 2017 Nov 13;17:204. doi: 10.1186/s12909-017-1031-z (PMC5683520; doi:10.1186/s12909-017-1031-z)
Supplement: Additional file 1: — Human Resource Research in Healthcare – Questionnaire. (PDF 224 kb) [file 12909_2017_1031_MOESM1_ESM.pdf]

Human Resource Research in Healthcare - Questionnaire

Semmelweis University, Health Services Management Training Centre (EMK),
Hungarian Medical Residents' Association (MRSZ), 2008.

Dear Colleague,

During our survey study we would like to collect information about the Hungarian medical doctors' opinion and situation, to support the elaboration of a healthcare human resources strategy. Please answer all of the following questions! Filling in the questionnaire assists our work and contributes to achieving our goal that is improving your working conditions.

Most of the questions require the selection of one answer. Where multiple answers are allowed, it is noted. Filling in the questionnaire is voluntary and anonymous. The data will be analysed only aggregated by statistical methods. If you have any question, comment or suggestion please contact us using the following e-mail address: girasek@emk.sote.hu.

Thank you for your cooperation!

Budapest, 16th November, 2007

The research team:
Miklós Szócska MD, director EMK
Edit Eke MD, EMK
Edmond Girasek, EMK
Emília Ilona Gaál, MRSZ

Participation is voluntary

A1. Are you …
1 – Male,
2 – Female?

A2. What is your citizenship? *(If you are not a Hungarian citizen, please indicate your native language)*

____

A3. Your year of birth? [_][_][_][_]

A4. What is your marital status?
1 – Single
2 – Cohabitation
3 – Married
4 – Divorced

A5. Do you have children?
0 – No
1 – Yes → How many children do you have: _____

A6. Please fill in the following table. *At the type of settlement, please use the following categories: 1 – Budapest, 2 – County town, 3 – Town, 4 – Village, 5 -- Abroad*

	Type of settlement	Postal code	County
Your residency before the university	1 – 2 – 3 – 4 – 5		
Your permanent residency	1 – 2 – 3 – 4 – 5		
Your temporary residency	1 – 2 – 3 – 4 – 5		
Place of work	1 – 2 – 3 – 4 – 5		

A7. What is the highest educational level of your father?
1 – Elementary school
2 – Vocational training
3 – High school graduation
4 – Technical school
5 – College degree
6 – University degree
7 – Ph.D. or other scientfic degree

A8. What is the highest educational level of your mother?
1 – Elementary school
2 – Vocational training
3 – High school graduation
4 – Technical school
5 – College degree
6 – University degree
7 – Ph.D. or other scientific degree

The research project was made possible by a grant (OFA/7341/0032) from the Hungarian National Employment Foundation (Országos Foglalkoztatási Közalapítvány).

A9. Please fill in the following table by assessing your language skills. Please answer according to **your subjective assessment!** *(Select the appropriate number in each cell: 0 = not at all, 5 = native level. Please add other languages, if applicable!)*

**Language**	**Speaking**	**Listening**	**Reading**	**Writing**
English	0 – 1 – 2 – 3 – 4 – 5	0 – 1 – 2 – 3 – 4 – 5	0 – 1 – 2 – 3 – 4 – 5	0 – 1 – 2 – 3 – 4 – 5
German	0 – 1 – 2 – 3 – 4 – 5	0 – 1 – 2 – 3 – 4 – 5	0 – 1 – 2 – 3 – 4 – 5	0 – 1 – 2 – 3 – 4 – 5
	0 – 1 – 2 – 3 – 4 – 5	0 – 1 – 2 – 3 – 4 – 5	0 – 1 – 2 – 3 – 4 – 5	0 – 1 – 2 – 3 – 4 – 5
	0 – 1 – 2 – 3 – 4 – 5	0 – 1 – 2 – 3 – 4 – 5	0 – 1 – 2 – 3 – 4 – 5	0 – 1 – 2 – 3 – 4 – 5

A10. You are a...

1 – First-year medical resident
2 – Second-year medical resident
3 – First-year medical resident, but already have one specialisation
4 – Second-year medical resident, but already have one specialisation
5 – Other

A11. What is your current field of specialisation?

____

A12. Do you have a medical specialisation?

1 – No, I am preparing for my first one
2 – Yes, I have one
3 – Yes, I have more

A13. If you already have one or more medical specialisation, what are these?

1. ____________________________________
2. ____________________________________
3. ____________________________________

A14. Which program did you graduate in?

1 – General Medicine
2 – Dentistry

A15. Which year did you graduate?

[ ][ ][ ][ ]

A16. At which university did you graduate?

1 – University of Debrecen
2 – University of Pécs
3 – Semmelweis University
4 – University of Szeged
5 – Foreign University

A17. During the residency which university do you belong to?

1 – University of Debrecen
2 – University of Pécs
3 – Semmelweis University
4 – University of Szeged

A18. Do/did you participate in PhD studies?

1 – No, and I do not intend to
2 – No, but I am thinking about it
3 – Yes, I am PhD student
4 – I have PhD degree

A19. If you participate/d in PhD studies, what is/was your main motivation?

1 – Professional career
2 – Research interest
3 – Forced to (e.g. clinical job, management position)
4 – Other

A20. Would like to work as a specialist?

1 – Yes, in a job corresponding to my specialisation to be obtained now
2 – Yes, in a job corresponding to my former specialisation(s)
3 – Yes, but in a different field
4 – No, but I would like to work in healthcare (e.g. pharmaceutical company) in the following field: ____
5 – No, I do not want to work in healthcare, rather in the following field: ____________________

A21. Where would you like to work?

County	City	1 – general practice<br>2 – outpatient care<br>3 – inpatient care<br>4 – other in healthcare<br>5 – other, not in healthcare

The research project was made possible by a grant (OFA/7341/0032) from the Hungarian National Employment Foundation (Országos Foglalkoztatási Közalapítvány).

A22. If you do not get an appropriate job in a preferred city (A21), would you be willing to commute to another city?

1 – Yes

2 – No

A23. If you do not get an appropriate job in a preferred city (A21), would you be willing to move to another city?

1 – Yes, I am interested in any opportunities, even with a change of specialisation.

2 – Yes, if I get support (accommodation, mortgage with preferential rates), even with a change of specialisation.

3 – Yes, but only in a job corresponding to my current specialisation.

4 – Yes, but only in a job corresponding to my current specialisation, only if I get support.

5 – No, in this case I would consider other opportunities. Please specify: ______________________

A24. Have you spent one or more months abroad for **professional purpose**?

1 – No → ***Please, go to question A29.***

2 – Yes → for a total of ________ months

A25. How many times have you been abroad for **professional purposes**?

[__][__] times

A26. Which countries have you been to for **professional** purposes?

____

A27. What kind of status did you have abroad?

1 – Medical researcher

2 – Practising medical doctor

3 – Medical student

4 – As a relative

5 – Other

A28. Please rate your **professional** experience abroad. *(rate on a 5-grade scale: 1 = very bad, 5 = very good)*

1 – 2 – 3 – 4 – 5

A29. Do you plan to work abroad?

1 – No → ***Please, go to question C1.***

2 – Yes →

1 – Not as a medical doctor → ***Please, go to question D1.***

2 – As a medical doctor in my profession →***Please continue the questionnaire here below***

B1. Have you already taken any steps to work abroad?

1 – Yes, I have a written agreement with an institution abroad

2 – Yes, I have an oral agreement with an institution abroad

3 – Yes, I am in correspondence, telephone connection with one/more potential institution(s), and/or I have been to job interview(s) at one or more potential institution(s)

4 – I have no agreement yet with an institution abroad, but I am looking for the opportunity

5 – I have already inquired about the conditions and possibilities

6 – This is in my future plan, but I am not looking for opportunities actively now.

B2. Where did/would you obtain information to realise your plan of working abroad? *(**Multiple answers are possible**)*

1 – Direct connection with the institution abroad

2 – Recruitment company/companies in Hungary

3 – Recruitment company/companies abroad

4 – Professional organisations (chambers, associations)

5 – Internet

6 – Other, looking ads (e.g.: press)

7 – Friend(s)

The research project was made possible by a grant (OFA/7341/0032) from the Hungarian National Employment Foundation (Országos Foglalkoztatási Közalapítvány).

B3. Please evaluate the following items according to the extent they influenced your plan to work abroad *(1 = not at all, 5 = definitive influence! **Please rate each item**).*

Working environment	1 – 2 – 3 – 4 – 5	Professional opportunities	1 – 2 – 3 – 4 – 5
Salary	1 – 2 – 3 – 4 – 5	Conditions of scientific work	1 – 2 – 3 – 4 – 5
Quality of life	1 – 2 – 3 – 4 – 5	Social prestige	1 – 2 – 3 – 4 – 5
Learning a foreign language	1 – 2 – 3 – 4 – 5	Desire for adventure	1 – 2 – 3 – 4 – 5
Workload	1 – 2 – 3 – 4 – 5	Successful examples	1 – 2 – 3 – 4 – 5
Family influence	1 – 2 – 3 – 4 – 5	Informal payment in Hungary	1 – 2 – 3 – 4 – 5
Organisational circumstances	1 – 2 – 3 – 4 – 5	Future prospects of the Hungarian healthcare	1 – 2 – 3 – 4 – 5

B4. Which are the main limiting factors in working abroad? ***(Multiple answers are possible, please select the three most relevant options)***

1 – Limitations from the destination country (i.e., visa, equivalency of diploma)
2 – Integration difficulties in the destination country
3 – Difficulties in settling abroad
4 – Integration difficulties in the foreign professional community
5 – General difficulties
6 – Lack of or limitation in language skills

B5. When do you plan to start working abroad?

1 – As soon as possible, even before obtaining the specialty
2 – Immediately after obtaining the specialty
3 – Later, after obtaining the specialty

B6. How long do you plan to work abroad?

1 – Occasionally, while keeping my job in Hungary (e.g. weekend shifts, commuting)
2 – Occasionally, but only working abroad
3 – Short-term (less than 2 years)
4 – Medium-term (2-5 years)
5 – Long-term (more than 5 years)
6 – Life-long
7 – Depends on the opportunities and the circumstances

B7. What is your **current** plan regarding **returning to Hungary**?

1 – I will return after a few months for sure
2 – I would like to return within a few months
3 – I will return after a few years practice for sure
4 – I would like to return after a few years practice
5 – I will never return to Hungary for sure
6 – I would not like to return to Hungary
7 – Depends on the opportunities and the circumstances

B8. After returning to Hungary, where would you like to work?

1 – To my previous position at my previous workplace
2 – To a new position at my previous workplace
3 – To a new workplace, because my previous one will not be available anymore
4 – To a new workplace, because I do not prefer my previous one
5 – I would like to search for a job, because currently I do not have any
6 – I will return only, if I have an appropriate offer

The research project was made possible by a grant (OFA/7341/0032) from the Hungarian National Employment Foundation (Országos Foglalkoztatási Közalapítvány).

B9. In which countries are you planning to work? *(If you only have one in mind please indicate that one)*

**1. First choice**	
**2. Second choice**	
**3. Third choice**	

B10. What do you expect from your workplace abroad **(B9)**? Please rate the followings on a five-point scale *(1=worse than the current; 5=better than the current)***:**

Salary	1 – 2 – 3 – 4 – 5
Working conditions	1 – 2 – 3 – 4 – 5
Professional opportunities	1 – 2 – 3 – 4 – 5
Conditions of research	1 – 2 – 3 – 4 – 5
Carrier advancements	1 – 2 – 3 – 4 – 5
Prestige	1 – 2 – 3 – 4 – 5

B13. Are the administrative requirements of working abroad well-known for you?

1 – No, I have not looked for any information yet
2 – Yes, I heard about it, but have not looked for it myself
3 – Yes, I have all the information, but have not started the process yet
4 – Yes, I have all the information and started the process
5 – Yes, I could start work abroad immediately

B14. What are the most important sacrifices you are prepared to make to work abroad? ***(Multiple answers are possible, please select maximum three options, which are most relevant for you)***

1 – Improving my language skills
2 – Learning a new language
3 – Obtaining a required training abroad
4 – Obtaining a required training about the operation of the healthcare system
5 – Taking a job below my qualification
6 – Taking a position below my qualification
7 – Changing my field of specialization
8 – Being away from my family and friends

Please, go to D1.

C1. This section is to be completed only by those, who do not plan to work abroad.
How much do the following factors have contributed to your decision of not planning to work abroad? *(1 = not at all, 5 = definitive influence! **Please rate each item**)*

Regulations (visa, diploma recognition)	1 – 2 – 3 – 4 – 5	I cannot imagine myself anywhere else	1 – 2 – 3 – 4 – 5
Prestige of medicine and health professionals	1 – 2 – 3 – 4 – 5	Opportunities in my profession	1 – 2 – 3 – 4 – 5
Working conditions	1 – 2 – 3 – 4 – 5	Lack of language skills	1 – 2 – 3 – 4 – 5
Family matters	1 – 2 – 3 – 4 – 5	Fear of failure	1 – 2 – 3 – 4 – 5
Integration and adaptation difficulties	1 – 2 – 3 – 4 – 5	Opportunities are better in Hungary	1 – 2 – 3 – 4 – 5
Informal payment in Hungary	1 – 2 – 3 – 4 – 5	Conditions of professional work	1 – 2 – 3 – 4 – 5
	1 – 2 – 3 – 4 – 5	Organizational structure	1 – 2 – 3 – 4 – 5

The research project was made possible by a grant (OFA/7341/0032) from the Hungarian National Employment Foundation (Országos Foglalkoztatási Közalapítvány).

I feel responsibility towards Hungarian patients and healthcare		Future of the Hungarian system	1 – 2 – 3 – 4 – 5
Proximity of friends and family	1 – 2 – 3 – 4 – 5	I would like to stay home	1 – 2 – 3 – 4 – 5

C2. What do you think about the following factors, how much do they influence the intentions of working abroad? *(1 = not at all, 5 = definitive influence! **Please rate each item**)*

Working environment	1 – 2 – 3 – 4 – 5	Professional opportunities	1 – 2 – 3 – 4 – 5
Salary	1 – 2 – 3 – 4 – 5	Conditions of scientific work	1 – 2 – 3 – 4 – 5
Quality of life	1 – 2 – 3 – 4 – 5	Social prestige	1 – 2 – 3 – 4 – 5
Learning a foreign language	1 – 2 – 3 – 4 – 5	Desire for adventure	1 – 2 – 3 – 4 – 5
Workload	1 – 2 – 3 – 4 – 5	Successful examples	1 – 2 – 3 – 4 – 5
Family influence	1 – 2 – 3 – 4 – 5	Informal payment in Hungary	1 – 2 – 3 – 4 – 5
Organisational circumstances	1 – 2 – 3 – 4 – 5	Future prospects of Hungarian healthcare	1 – 2 – 3 – 4 – 5

Please answer the following questions regardless of your own ambitions and intentions of working abroad.

D1. Upon applying to medical training what was your aim after graduation?

1 – Medical doctor, without concrete idea
2 – Medical doctor, who cures patients (practitioner)
3 – Medical researcher
4 – Working with medical diploma outside healthcare (e.g.: pharma industry, public health authority)
5 – Medical doctor, who cures patients (practitioner), but abroad
6 – Working in non-medical profession
7 – I applied because I had no better idea
8 – I did not want to be a medical doctor, I applied to this university solely due to the pressure of my family

D2. Please evaluate the following items according to the extent they influenced your choice of medical career *(1 = not at all, 5 = definitive influence! **Please rate each item**).*

Focus of my interest	1 – 2 – 3 – 4 – 5	Informal payment	1 – 2 – 3 – 4 – 5
I wished to be a „doctor”	1 – 2 – 3 – 4 – 5	Helping profession	1 – 2 – 3 – 4 – 5
Scientific career	1 – 2 – 3 – 4 – 5	Intellectual work	1 – 2 – 3 – 4 – 5
This diploma was needed to my goals	1 – 2 – 3 – 4 – 5	Social utility of the medical profession	1 – 2 – 3 – 4 – 5
Opportunities to work abroad	1 – 2 – 3 – 4 – 5	Good job opportunities	1 – 2 – 3 – 4 – 5
Salary	1 – 2 – 3 – 4 – 5	Sense of vocation	1 – 2 – 3 – 4 – 5
Social Prestige	1 – 2 – 3 – 4 – 5	I felt talent for this profession	1 – 2 – 3 – 4 – 5
Family influence	1 – 2 – 3 – 4 – 5	Other: _________________	1 – 2 – 3 – 4 – 5

D3. Please evaluate the following items according to the extent they influenced your choice of specialization *(1 = not at all, 5 = definitive influence! **Please rate each item**).*

Focus of my interest	1 – 2 – 3 – 4 – 5	It just happened this way	1 – 2 – 3 – 4 – 5
Professional challenges	1 – 2 – 3 – 4 – 5	Prestige among medical doctors	1 – 2 – 3 – 4 – 5
Scientific career	1 – 2 – 3 – 4 – 5	Informal payment	1 – 2 – 3 – 4 – 5
Opportunities to work abroad	1 – 2 – 3 – 4 – 5	Innovation opportunities	1 – 2 – 3 – 4 – 5
Family influence	1 – 2 – 3 – 4 – 5	Social prestige	1 – 2 – 3 – 4 – 5
Lifestyle	1 – 2 – 3 – 4 – 5	Relation with patients	1 – 2 – 3 – 4 – 5
Salary	1 – 2 – 3 – 4 – 5	Good job opportunities	1 – 2 – 3 – 4 – 5

The research project was made possible by a grant (OFA/7341/0032) from the Hungarian National Employment Foundation (Országos Foglalkoztatási Közalapítvány).

D4. What do you think the desired optimal **net salary** for doctors in Hungary should be, so that they do not have to look for additional income (from informal payment and/or from second, third jobs) and they would not considering leaving the medical profession or working abroad?

**1. Medical doctors** (in practice, first or second year resident doctors)?	HUF
**2. Medical doctors before the specialty exam** (after obtaining 2 years of residency)?	HUF
**3. Specialists**?	HUF

D5. If you could choose again, would you choose …

	**Yes**	**No**
the medical profession?	1	2
the same residency programme at the same university?	1	2
the same specialisation?	1	2

D6. Would you like to receive information about the research results?

1 – Yes
2 – No

Thank you for your participation!

In the next phase of the research we plan to carry out structured personal and focus group interviews. If you are available to participate and willing to share your experiences and views, please provide your contact details below.

Data and any personal information will be handled confidentially in line with privacy regulations. Personal data will not be made available to any third parties. Personal data will be treated separately form the questionnaires, and will not be used to identify individual respondents.

Name:

Phone:

E-mail:

The research project was made possible by a grant (OFA/7341/0032) from the Hungarian National Employment Foundation (Országos Foglalkoztatási Közalapítvány).
